# Transforming Waste into Value: The Role of Recovered Carbon Fibre and Oil Shale Ash in Enhancing Cement-Based Structural Composites

**DOI:** 10.3390/ma18245636

**Published:** 2025-12-15

**Authors:** Regina Kalpokaitė-Dičkuvienė, Inga Stasiulaitiene, Arūnas Baltušnikas, Samy Yousef

**Affiliations:** 1 Laboratory of Materials Research and Testing, Lithuanian Energy Institute, Breslaujos 3, LT-44403 Kaunas, Lithuania; regina.kalpokaite-dickuviene@lei.lt (R.K.-D.);; 2 Department of Environmental Technology, Faculty of Chemical Technology, Kaunas University of Technology, LT-50254 Kaunas, Lithuania; 3 Department of Production Engineering, Faculty of Mechanical Engineering and Design, Kaunas University of Technology, LT-51424 Kaunas, Lithuania

**Keywords:** cement-based structural composites, metakaolin, oil shale ash, recycled carbon fiber, waste wind turbine blades

## Abstract

Economic and technological factors necessitate the use of alternative fuels during oil shale combustion, a process that generates substantial amounts of solid waste with varying ash compositions. This study evaluates the potential of two such waste materials: (i) fly ash derived from the combustion of oil shale (a fine particulate residue from burning crushed shale rock, sometimes combined with biomass), and (ii) short carbon fibres recovered from the pyrolysis (a process of decomposing materials at high temperatures in the absence of oxygen) of waste wind turbine blades. Oil shale ash from two different sources was investigated as a partial cement replacement, while recycled short carbon fibres (rCFs) were incorporated to enhance the functional properties of mortar composites. Results showed that carbonate-rich ash promoted the formation of higher amounts of monocarboaluminate (a crystalline hydration product in cement chemistry), leading to a refined pore structure and increased volumes of reaction products—primarily calcium silicate hydrates (C–S–H, critical compounds for cement strength). The findings indicate that the mineralogical composition of the modified binder (the mixture that holds solid particles together in mortar), rather than the fibre content, is the dominant factor in achieving a dense microstructure. This, in turn, enhances resistance to water ingress and improves mechanical performance under long-term hydration and freeze–thaw exposure. Life cycle assessment (LCA, a method to evaluate environmental impacts across a product’s lifespan) further demonstrated that combining complex binders with rCFs can significantly reduce the environmental impacts of cement production, particularly in terms of global warming potential (−4225 kg CO_2_ eq), terrestrial ecotoxicity (−1651 kg 1,4-DCB), human non-carcinogenic toxicity (−2280 kg 1,4-DCB), and fossil resource scarcity (−422 kg oil eq). Overall, the integrative use of OSA and rCF presents a sustainable alternative to conventional cement, aligning with principles of waste recovery and reuse, while providing a foundation for the development of next-generation binder systems.

## 1. Introduction

The cement industry is a major partner in European construction activities. This is evident in the large European cement market, valued at over USD 19.02 billion in 2023. It had a production volume of 181.17 million metric tons in 2022. With future development ambitions, the European market is expected to reach USD 31 billion by 2033 and 202.83 million metric tons by 2028 [1,2]. However, high cement production leads to large carbon dioxide emissions, estimated at 7% of global emissions and 4% within the European Union [3]. The climate impacts per ton range from 832 to 1075 kg CO_2_-equivalents [4]. In addition to CO_2_, cement production releases hydrochloric acid, fluorhydric acid, and air pollutants. These emissions cause acid rain [5,6,7]. To address these challenges, major European cement producers plan to use more renewable electricity and partially replace cement in building materials [8]. In this context, oil shale ash (OSA) has recently been utilised as a substitute for cement. OSA is produced by burning oil shale, which generates approximately 40–70% ash, a significant amount in the context of pollution [9]. This is due to its silicon dioxide (SiO_2_) and aluminium oxide (Al_2_O_3_) content, which reacts with calcium hydroxide (Ca(OH)_2_) from cement hydration. This reaction forms more calcium silicate hydrates (CSH), improving the physical and mechanical properties of cement matrices [10,11,12]. OSA is widely available as a solid residue from oil shale combustion, with limited engineering uses or usually landfilled [13]. Using OSA as a building material helps reduce environmental problems and increases its economic value [14]. Accordingly, OSA has been used as a partial cement substitute in concrete, cement paste, mortar, and pavement [15,16,17,18]. Results showed that adding 10–30% OSA to composites slightly lowers their performance compared to cement-based materials. Reductions are in the ranges of 18–42% for compressive strength, 27–37% for splitting strength, 3–61% for flexural strength, and 3–61% for modulus of elasticity [10]. Additional supplementation is needed to offset these reductions.

Metakaolin (MK) is a supplementary material rich in silicate and aluminate, promoting pozzolanic reactions [19]. It improves pore structure and reduces permeability and porosity, resulting in enhanced mechanical strength, durability, and corrosion resistance. MK is synthesised from natural sources [20,21,22]. When incorporated into the binder, MK reacts with portlandite (CH) to generate additional CSH through a synergistic effect [23,24]. Despite OSA’s and MK’s strong environmental and sustainability potential, their elasticity needs improvement [25,26]. Reinforcing cementitious composites with short fibres is common for this purpose. Many fibre types are used, classified as synthetic or natural fibres (like wood) [27,28,29,30,31,32]. Among them, synthetic carbon fibres have high strength, elasticity, and thermal and chemical stability. These properties slow down failure and improve mechanical performance [33]. However, their high cost limits widespread adoption [34]. Alternatives must be found to produce or recycle them more cheaply. Thus, recycled fibres from fibre-reinforced polymer (FRP) waste are gaining attention. They are less expensive and offer acceptable performance [35,36,37]. However, cementitious matrices made with recycled wet fibres (resin-blended) degrade over time. This is due to the alkaline activity of cement, which affects long-term performance [38,39]. Therefore, the resin part of recycled wet fibres should be removed before using them as fillers. The pyrolysis process has recently been utilised to decompose the resin in FRP waste [40,41]. It has proven highly efficient.

Wind turbine blade waste (WTB) consists of FRP with high fibre content, about 70 wt.%. Its abundance is estimated at 43 million tons by 2050 [42,43]. Pyrolysis and its catalytic process can decompose WTB resin into high-value chemicals, such as phenol and aromatics [44,45,46]. The fibres remain as solid residue due to high thermal resistance. The purity of recycled fibres improves after water washing and oxidation. Studies show this method recovers pure glass and carbon fibres. These fibres enhance compressive strength and reduce mortar absorption and water content [47,48,49]. Thus, WTB offers a cheap, renewable source for short carbon fibres (rCFs) via pyrolysis with post-treatment oxidation. This study aims to enhance the environmental and mechanical properties of cement mortar by incorporating eco-friendly additives. It uses OSA, MK, and rCFs from WTB pyrolysis. The effects on hydration phase composition, pore size, specific surface area, compressive and flexural strength, freeze–thaw cycling, and water absorption were observed over a period of up to 120 days. The study also evaluated the environmental performance of advanced composites compared to conventional materials.

## 2. Experimental

### 2.1. Materials

In this research, Portland cement (PC: CEM Grade I 42.5R) conforming to EN 197-1 [50] was used. MK powder was obtained by calcining kaolin at 800 °C for 2 h. Two batches of oil shale ash (OSA) were supplied by the Eesti and Auvere power plants in Estonia [9,51]. Ashes were taken from electrostatic precipitators [9]. Sand in a (0.0–2.0) mm fraction and a superplasticiser (conforming to EN934-3 [52]) were also used. Detailed information on the chemical and phase composition of the raw oil shale can be found in [9,53]. At Eesti Plant, OSA1 was produced by burning only oil shale. This OSA had a high sulfate (CaSO_4_) content and a specific gravity of 2946 kg/m^3^. At Auvere Plant, OSA2 with a high carbonate (CaCO_3_) content and a specific gravity of 2756 kg/m^3^ was produced by burning oil shale mixed with biomass [9]. The oxide composition of PC, MK, and OSA binders is summarised in Table 1 [9,51,53]. According to our previous study [9], both ashes contain calcium-bearing minerals, such as anhydrite, larnite, calcite, lime, and portlandite, together with a high content of orthoclase and quartz. More detailed analysis is presented in [9]. The rCF used in this study was extracted from WTB, which is composed of carbon fibre-reinforced unsaturated polyester resin, through pyrolysis and post-treatments. The pyrolysis process was performed on chopped WTB at 500 °C in a laboratory reactor to decompose the resin fraction. Afterwards, rCF was separated and purified from the solid residue by sieving and oxidation in air at 450 °C as detailed in [47].

### 2.2. Design of the Experiments

**Figure 1:**
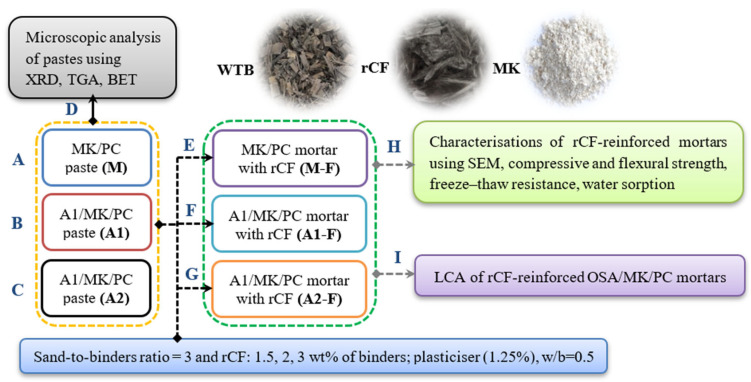
Flowchart of the present work, including the sample preparation and characterisation.

Figure 1 presents the current workflow diagram for the composite preparation and characterisation in this research. Usta et al. 2020 highlighted the potential of OSA as a binder [11], prompting the use of 6% MK and 20% OSA as cement replacements in this study. The control sample (M) used PC (94%) and MK (6%) to assess the effect of the two ashes. The process began with preparing cement pastes (water-to-cement ratio of 0.5) using various binders (A–C), omitting inert rCF and sand to isolate the impact of OSA on hydration products via microscopic analysis (D). Next, sand and rCF were added in different proportions to create rCF-reinforced OSA/MK/PC mortars (E–G) to study their mechanical properties (H). Finally, the environmental performance of the composites was assessed (I). Each stage will be detailed in the following sections, in the order listed below.

### 2.3. Preparation of Composites

To prepare the cement mortars, rCF (F: 1.5, 2, and 3 wt.% of binders) and a sand-to-binder ratio of 3 were added to the homogeneous mixtures of M, A1, and A2. This produced M-F, A1-F, and A2-F mortars. The rCF ratio was chosen based on the most common ratio used in the literature [54,55]. Since OSA and MK consume more water than cement [10,53,56], a superplasticiser was used to keep the slump within the 155–170 mm range. The M-F mortars were prepared by mixing PC, MK, and sand particles using a planetary mixer for 5 min (dry mixing). Then, water and plasticiser were added, and mixing continued for another 5 min (wet mixing). After that, rCF was added gradually with the specified content. Mixing continued for an additional 5 min at a slow speed. This step helped distribute the fibres evenly without breaking or damaging their surfaces [48]. The homogeneous mixture was poured into cubic (20 × 20 mm) and prismatic (20 × 20 × 100 mm) metal moulds and then shaken. These samples were prepared for compressive and flexural tests [57]. The moulds were cured for 24 h, followed by hydration for 28 and 120 days in lime water. This prevented additional carbonation and ensured consistent results across samples [58]. A1-F and A2-F mortars were made using the same procedures as M-F mortar. However, for these, the binder OSA was added to PC and MK and mixed under dry conditions first. Fibres were added in wet conditions, followed by the preparation of specimens for compressive and flexural tests as described above. After 28 days of hydration, one set of samples underwent freeze–thawing at temperatures of (−18 + 23 °C) [9]. Other samples were tested for mechanical strength and water absorption. Finally, each sample was given a code based on its composition, as shown in Table 2.

### 2.4. Characterizations of the Fabricated Cement Composites

The effect of different OSAs on the crystalline phases and hydration products of cement paste was investigated using X-ray diffraction (XRD) with a BRUKER D8 ADVANCE diffractometer (Bruker, Germany). The XRD analysis was performed on A1 and A2 pastes for up to 120 days. The content of hydration products and chemically bound water in the pastes was determined using thermogravimetric analysis (TGA: LINSEIS STA PT1600, (Linseis, Germany) in nitrogen ambient at 20 °C/min. The specific surface area and pore size of the pastes were measured using a Brunauer–Emmett–Teller (BET) apparatus AUTOSORB-iQ-K/MP (Quantachrome, USA) based on the nitrogen physisorption approach. The deposition of hydration products on rCF surfaces was investigated using scanning electron microscopy (SEM). The effect of OSA composition type and rCF concentration on the mechanical strength (compressive and flexural) of cement mortars was investigated using a Zwick Roell universal testing instrument (ZwickRoell group, Germany) at a rate of 0.5 mm/min, in accordance with the European guidelines EN 196-1 [59]. The effect of these additives on the water absorption of mortars was tested according to EN 1015-18 standard [48,60]. The sorptivity (A) of the mortars was determined by plotting the linear relationship between capillary water absorption (i) and the fourth root of time (t0.25) for each sample. The slope was calculated to estimate the value of A using the formula (i = A t0.25) [9]. The frost resistance test of the mortars was performed in accordance with LST L 1413.11 standard [61]. The hydrated specimens were taken from saturated lime water, air-dried for 5 min, placed in a freezer at −17 °C for 4 h, and then immersed in a bath of tap water at 20 °C for 20 h. This freeze–thaw (F-T) cycle was repeated 25 times.

### 2.5. Life Cycle Assessment of rCF-Reinforced OSA/MK/PC Mortar

**Figure 2:**
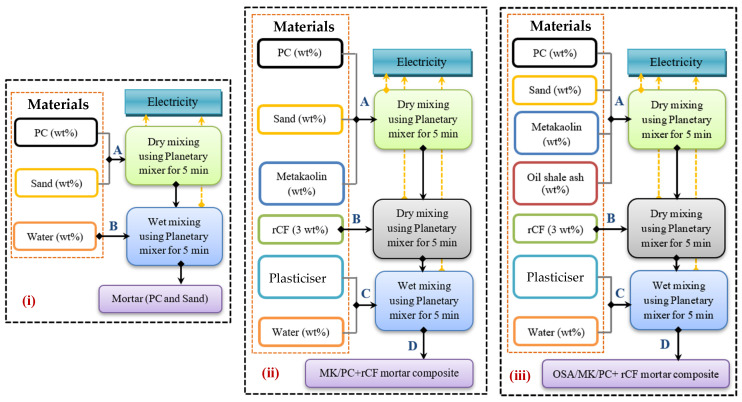
LCA layout of (**i**) mortar, (**ii**) OSA/MK/PC mortar composite, and (**iii**) OSA/MK/PC + rCF mortar composite production scenarios.

In this section, the impact of using MK and OSA as partial cement substitutes and rCF as a reinforcing material for cement mortar on its environmental performance was studied. The evaluation was conducted using a life cycle analysis (LCA) with SimaPro software. The analysis followed ISO 14040 and 14044 standards, using a cradle-to-gate approach [62]. Based on these guidelines, the goal was to study the potential environmental impacts of rCF-reinforced OSA/MK/PC mortar production. The study considered all stages of the production process, required materials, and electricity. The functional unit (FU) was defined as the production of 1 m^3^ of rCF-reinforced OSA/MK/PC mortar within the European region, which served as the geographical context [63]. This study focused solely on mortar composite production, excluding the construction and end-of-life phases. Inventory data for the evaluation process were gathered from experimental results obtained during preparation and characterisation. These experiments produced mortar composites with maximum strength. Data for main materials—PC, MK, sand, OSA, rCF, water, and plasticiser—were extracted directly from SimaPro’s default Ecoinvent database. Both types of OSA were defined in the LCA based on their composition, listed in Table 1, and MK was defined as calcined kaolin. Because rCF is not available in the Ecoinvent database and only CF-reinforced polymer is, rCF was defined as graphite (its main component). According to the results, A1-3F (with high sulfate content) and A2-3F (with high carbonate content) provided the highest strength. The compositions of both were considered the main scenarios and then compared with typical mortar and OSA/MK/PC mortar. Based on these scenarios, an LCA layout for each was constructed, along with the corresponding constraints, as shown in Figure 2. The evaluation continued by identifying the most frequent impact categories, which helped determine the technical and ecological potentials of the new composites. The study assumed that all materials used in production were available on-site, so transportation was not considered. Finally, input data, including materials and electricity consumption, were collected and are presented in Table 3 [64,65,66,67,68]. According to Diaz-Basteres [64], producing one ton of mortar consumes 4 kWh/ton for dry mixing and the same amount for wet mixing. The electricity required to produce 1 m^3^ of mortar composite was calculated in this research after adjusting for the specified functional unit.

## 3. Results and Discussions

### 3.1. XRD Analysis of OSA/MK Cement Pastes

**Figure 3:**
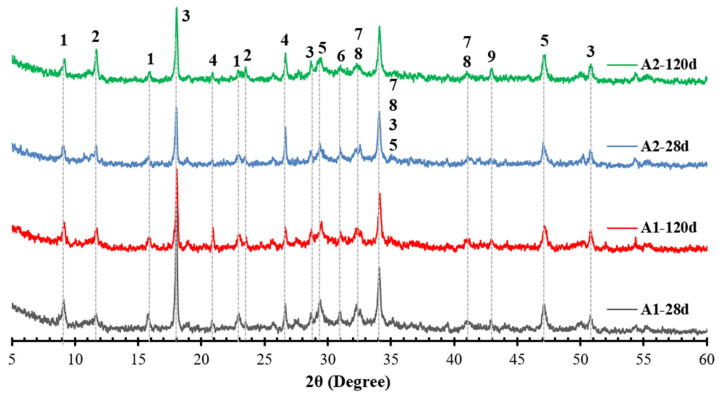
XRD of A1 and A2 pastes, composed of blended binder (OSA/MK/PC) after 28 and 120 hydration days. 1—AFt, 2—Mc, 3—CH, 4—SiO_2_ (Quartz), 5—CaCO_3_, 6—CaMg(CO_3_)_2_, 7—alite (C_3_S), 8—belite (C_2_S), and 9—MgO.

**Figure 4:**
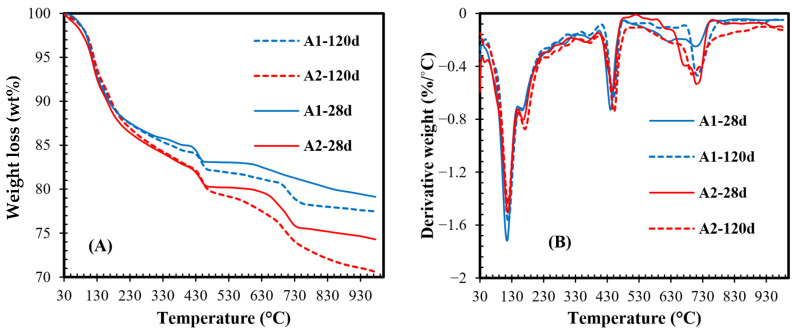
TG (**A**) and DTG (**B**) curves of A1 and A2 pastes, composed of blended binder (OSA/MK/PC), after 28 and 120 hydration days.

Since rCF is an inert material and does not participate in chemical reactions, the cement pastes without fibre and sand filler were analysed by X-ray after 28 and 120 days, and patterns are presented in Figure 3. As mentioned earlier, the A1 and A2 pastes were prepared with a complex binder consisting of ash, MK, and cement. As cement and OSA contain many minerals of similar origin [9], the mineralogical composition of Portland cement-MK-OSA composites differs little from that of conventional hydrated cement. Significant differences arise due to the introduction of ash minerals, such as CaO, Ca(OH)_2_, CaCO_3_, and CaSO_4_, involved in the hydration reactions. Cement substitution with ash, which possesses a higher CaSO_4_ content (>14%) [9], influences hydration, as an increase in CaSO_4_ content accelerates the alite (C_3_S) reaction. In contrast, the incorporation of ash, characterised by a higher CaCO_3_ and CaO content [9], may contribute to monocarboaluminate formation (Mc) if enough aluminate is present. Both ashes contain a low amount of aluminium, and most of it is present in a K-feldspar compound that is hardly soluble. Thus, it may participate in the hydration reactions only at a later stage. Therefore, pozzolanic materials, such as MK, are needed to activate the hydration reactions. Replacing PC with OSA does not change the overall mineralogical composition of the hydrated samples after 28 and 120 days (Figure 3). All cementitious phases, including AFt, Mc, CH, alite (C_3_S), belite (C_2_S), and calcite, together with silicon or magnesium oxides, are found in both compositions (A1 and A2 pastes). Nevertheless, the intensity of the phases, particularly AFt, Mc, and CH, differs significantly. Contrary to plain systems with ash [9], the incorporation of MK results in an increase in both AFt and Mc content, although their intensity changes throughout the hydration process. It was shown in [69,70,71] that the sulfate and calcite content both variably affect the hydration of clinker, leading to changes in AFt and Mc formation. It is known that the presence of calcite destabilises the AFm in favour of AFt and Mc [69], whereas a larger volume of AFt is stabilised by the presence of sulfate [69]. MK, on the other hand, consumes portlandite to produce an additional CSH phase, and Mc in the presence of calcite, if CH content is in excess [51,72,73]. Due to the presence of MK in the A1 system, which has a higher sulfate content, the content of AFt should increase, and Mc should decrease with time, as indicated in [74]. However, Figure 4 shows that the AFt content decreases in the A1 system, whereas it increases in the A2 system, which possesses a higher content of carbonates. The precipitation of ettringite requires additional calcium from the system, which may be supplied by portlandite through the hydration of C3S [74]. The lower peak of CH and increase in Mc in the A1 system after long-term hydration confirm observations presented in [74], that the alumina from metakaolin participates in the formation of ettringite until the gypsum and CaSO_4_ are depleted (Figure 3). On the contrary, the Mc and CH peaks increase throughout the hydration in the A2 system (Figure 3), confirming the synergistic effect of MK and calcite mentioned in [71]. The low intensity of peaks at 32–33° (2θ°) suggests the higher dissolution rate of C_3_S and/or C_2_S, thus explaining an increase in CH content in the A2 system after long-term hydration. Consequently, an excess of CH facilitates the MK reaction with carbonates, increasing Mc content.

### 3.2. Thermogravimetric Analysis of OSA/MK Cement Pastes

Figure 4 shows the TGA and derivative thermogravimetry (DTG) curves of the A1 and A2 pastes after 28 and 120 days. Based on these measured data, the content of the hydration products and chemically bound water was calculated [51] and is summarised in Table 4. As mentioned above, both types of OSA initially contain CH [9], which is one of the main hydration products of cement paste. This means that only a relative comparison can be made when evaluating changes in the assemblage of reaction products in compositions with ash. The TGA curves (Figure 4A) show that the mass losses up to 450 °C in both samples do not change significantly with increasing hydration time, while they increase in the higher temperature range. Dehydration of CSH and AFt occurs at temperatures of up to 170 °C, while Mc and CH decompose at approximately 190 °C and 450 °C, respectively (Figure 4B). The decomposition of carbonates occurs at a broader temperature range from 700 to 790 °C, whereas the broader peak of carbonates in A2 paste suggests the partial carbonation of hydrates or decomposition of less crystalline forms of carbonates [75]. The calculated data in Table 4 shows that the bound water increases over time for both samples, indicating the higher volume of reaction products, primarily CSH, formed (Figure 4B). The CSH is an amorphous material; therefore, it is not observed in the XRD patterns (Figure 3). According to the TGA results, both compositions give nearly the same CH content, even though the intensity of CH in XRD curves differs among samples after 28 days. Nevertheless, after long-term hydration, the XRD data correlate with the TGA data, as the CH content decreases in A1 paste and increases in A2 paste. Moreover, the CH content is lowest in A1 and highest in A2 (up to 49%). The content of CC is also higher in the A2 set (up to 63%) and nearly doubles after long-term hydration. According to the findings reported by Hargis et al. (2024) [76], calcite may form rhombohedral crystals less than 5 µm in size, contributing to the formation of a more compact structure. Since the volume of hydration products is higher in A2 paste, this suggests that the pore structure in A2 was affected more than that in A1. To verify this, the nitrogen physisorption technique was used to measure the size of pores in the range of up to 50 nm, representing small capillary pores.

### 3.3. Nitrogen Gas Physisorption Analysis of OSA/MK Cement Pastes

**Figure 5:**
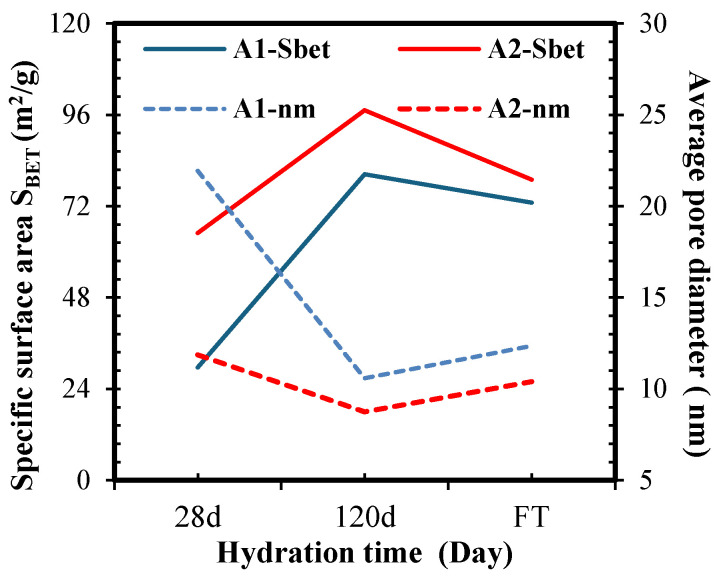
Effect of hydration time on pore size and specific surface area of A1 and A2 pastes, composed of blended binder (OSA/MK/PC).

Figure 5 shows the average pore diameter and specific surface area (SBET) of the A1 and A2 pastes. After short-term hydration (28 days), the pore diameter of the A1 paste becomes about twice that of the A2 paste. Over time, as long-term hydration (120 days) progresses, the difference between the samples decreases significantly. Notably, the average pore diameter in A2 (8.75 nm) is in the range of CSH gel pores [77], whereas for A1 it is slightly higher than 10 nm, which is characteristic of small capillary pores. Meanwhile, the specific surface area (SBET) increased by approximately 2.6 and 1.5 times for the A1 (80.4 m^2^/g) and A2 (97.2 m^2^/g) pastes, respectively. This increase correlates well with the TGA data, since the wb of A2 is higher than that of A1, implying the formation of a higher content of additional CSH in A2 due to chemical reactions with MK.

### 3.4. Morphology of rCF-Reinforced OSA/MK/PC Mortars

**Figure 6:**
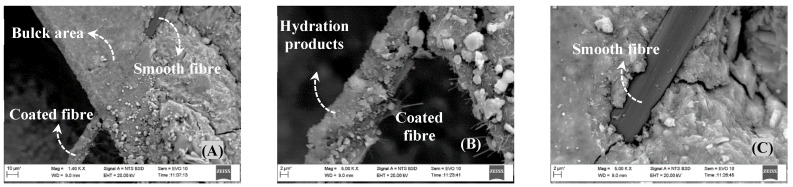
SEM images of the rGF interface in OSA/MK/PC mortar composites. (**A**) general view; (**B**,**C**) enlarged view of (**A**).

The SEM observation process was performed on the fracture surface of several rCF-reinforced OSA/MK/PC mortars. These mortars had different compositions of OSA and varying amounts of rCF. The scanning process focused on the surface features of the embedded fibres in the matrices. It was noticed that the fibres had the same features in all samples, regardless of the type of OSA and rCF content. Therefore, the most characteristic morphology is presented in Figure 6. SEM cross-section images of the interface between rCF and mortar binder (OSA/MK/PC) after 120 days of hydration (Figure 6A) reveal good integration of rCF in the bulk material. The surface of the fibre is completely covered with abundant hydration products, as shown in Figure 6B. In contrast, the upper side of the fibre is almost smooth (Figure 6C) due to the detachment of the hydration products during sample preparation. However, this indicates that the rCF (Figure 6B) acts as a crystallisation centre for hydration products. The adhesion to the fibre is weak due to the absence of chemical reactions between them [78,79]. The growth of hydration products on the rCF surface may contribute to reducing the micropore size, thus obstructing water ingress [9].

### 3.5. Compressive Properties of rCF-Reinforced OSA/MK/PC Mortars

**Figure 7:**
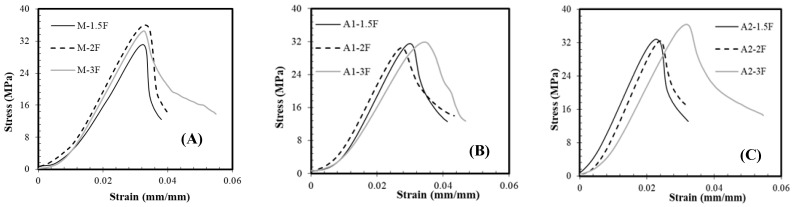
Typical stress–strain curves of mortars with different contents of rCF under uniaxial compression: (**A**), M-F (**B**), A1-F, and (**C**) A2-F mortars.

The typical uniaxial compressive stress–strain curves of the M-F, A1-F, and A2-F mortars after 28 and 120 hydration days are presented in Figure 7. As shown, the ascending part (elastic region) of the curves was approximately the same for all mortars, indicating that the type of binder and rCF do not affect the behaviour of the composite at this stage. When the curves deviate from linearity, the propagation of internal microcracks begins. Furthermore, it is clear that the higher the rCF content, the broader the descending part of the curve. This stress reduction indicates the coalescence of multiple microcracks into macrocracks, while the enhanced ductility behaviour is shown by compositions with higher rCF content [80,81]. Conversely, samples with lower rCF content show a steeper descending branch.

**Figure 8:**
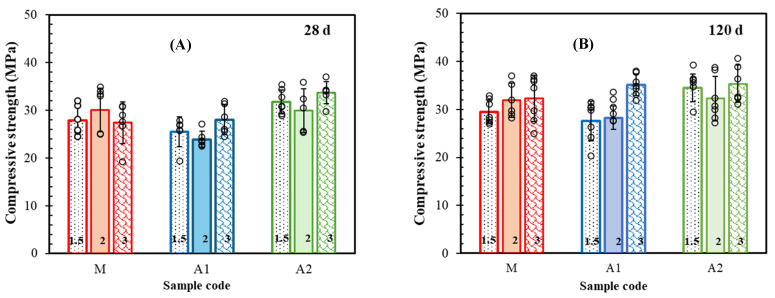
Impact of rCF content on compressive strength of mortars after (**A**) 28 days and (**B**) 120 days of hydration.

Based on the compressive stress–strain curves, the average compressive strengths of the M-F, A1-F, and A2-F mortars were calculated, and their values are summarised in Figure 8A, B after 28 and 120 days, respectively. As shown, the average compressive strength of the M-F, A1-F, and A2-F mortars after 28 days was estimated in the ranges of 28.5 MPa, 25.8 MPa, and 31.8 MPa, respectively. These values did not change significantly after a long-term hydration period and remained approximately in the same range, where the curing time and environment can contribute to the strength development only [57]. Additionally, the dependence of strength on fibre content is similar in systems with ash, although it is irreversible compared to the reference one (M). However, these values are lower than those of the reference paste without fibres (42.97 ± 1.42 MPa), indicating that rCF delays matrix hydration, a phenomenon also reported by Zhao et al. (2024) and Heo et al. (2020) [55,81]. Also, the A1-3F and A2-3F mortars (with higher rCF content) resulted in the highest strength (28.0 MPa and 33.8 MPa, respectively), contrary to the control mortar (M-F), reaching its highest strength with an intermediate content of rCF (30.1 MPa). Nonetheless, the A1-F group was obviously the weakest among the samples. Although the scattering of results within the groups suggests stress concentrations, possibly arising due to the nonuniform distribution of fibres [53,79], a continuous increase in strength with an increment of fibre content was observed after 120 days, as shown in Figure 8B. The increase in strength for the M group was around 15% and is consistent with results presented in [54,82,83]. The A1-F mortars showed the highest strength increment from 8 to 25% (up to 35.1 MPa), whereas the A2-F mortars demonstrated only a 5–9% increase (up to 35.3 MPa). The overall results reveal that the system with OSA, possessing a higher content of sulfates (A1), requires a longer hydration time to achieve a strength comparable to the reference mortar. Moreover, the A1 set is more sensitive to rCF content than the reference and A2 groups, thus implying that the impact of the binder’s phase composition prevails. Based on the TGA data (Table 3), the A2 composition contains a higher content of carbonates. The compressive strength of pure calcium carbonate is a few times lower than that of its mixture with aragonite due to the orthorhombic shape of aragonite crystals, the interlocking and interpenetration of which mainly contribute to the strength gain [76]. This implies that the development of strength in mortars with OSA depends mainly on the amount and transformation of hydrates that contribute to the refinement of the pore structure.

**Figure 9:**
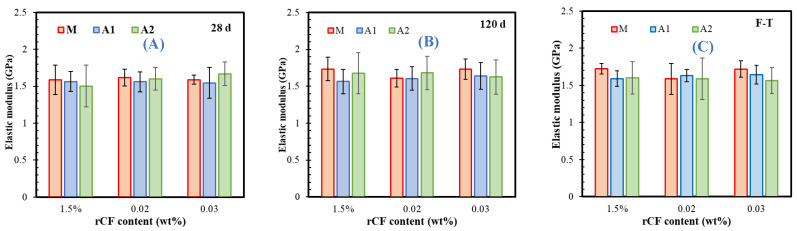
Effect of rCF content on elastic modulus of mortars after (**A**) 28 days, (**B**) 120 days, and (**C**) freeze–thawing process.

Finally, the elastic modulus of the M-F, A1-F, and A2-F mortars was obtained from the linear elastic phase of the compressive stress–strain curves and is displayed after 28 days (Figure 9A) and 120 days (Figure 9B) of hydration, and after freeze–thawing (F-T) (Figure 9C). The data show that both the hydration time and binder type only slightly affect elastic modulus, with values ranging from 1.5 to 1.7 GPa, likely because microfibres do not influence the elastic behaviour before crack formation. While the increased rCF content does not reveal a clear trend in the elastic modulus prior to F-T cycles, differences between samples become apparent after freeze–thawing (Figure 9C): A1-F mortars display a higher modulus than A2-F mortars. This suggests that the presence of OSA—associated with a higher sulfate content—contributes to a more rigid matrix, which may better resist material deterioration during freeze-thaw cycles.

### 3.6. Flexural Properties of rCF-Reinforced Mortars

**Figure 10:**
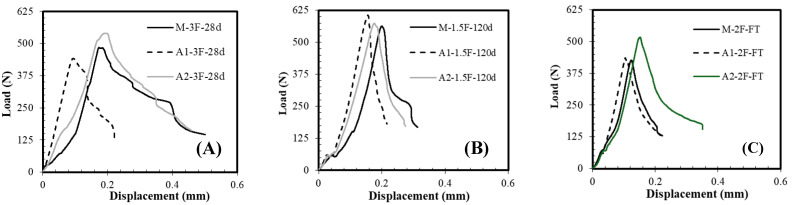
Typical load–displacement curves of mortars with different contents of rCF under flexure after (**A**,**B**) 28 and 120 days and (**C**) freeze–thawing.

**Figure 11:**
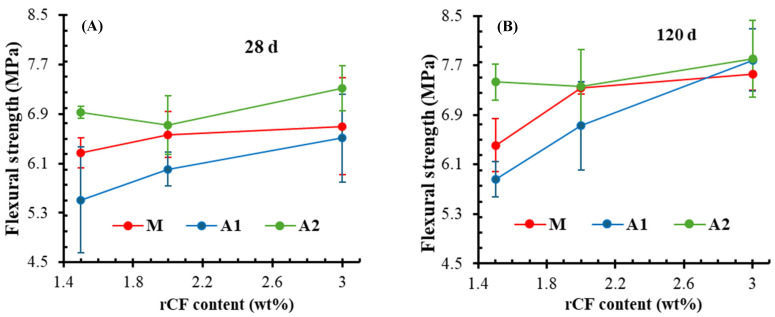
Impact of rCF content on flexural strength of mortars after (**A**) 28 and (**B**) 120 days.

Figure 10A,B present typical load–displacement curves in flexure for the M-F, A1-F, and A2-F mortars after 28 and 120 days, respectively. Flexure curves after freeze–thawing are also included (Figure 10C). As expected, the incorporation of a higher rCF content results in a better response to flexural load, increasing the displacement. The load peak increases with an increase in hydration time and reduces after freeze–thawing. The average flexural strength results after 28 and 120 days are presented in Figure 11. It is worth mentioning that the addition of commercial carbon fibres results in 6 to 9 MPa flexural strength after 28 days [82]. In this study, the application of rCF falls within this range, as the reference composition (M) yields a value of 6.5 MPa (Figure 11A). Regardless of the binder type, the strength of all compositions increases as the fibre content rises. The increment is relatively low, around 6% for the M and A2 compositions, whereas the A1 system, being the lowest, demonstrates the highest increase of 18%. The impact of rCF content on strength development becomes stronger after long-term hydration (Figure 11B), particularly in the A1 system, resulting in a 32% increase. On the contrary, the A2 composition demonstrates only a 5% increase, although it remains the strongest among all groups. It should be noted that in the A2 sample set, a slight reduction in flexural strength was observed at intermediate fibre content, likely due to the large scattering of the results, possibly caused by nonuniform distribution or poor bonding of fibres with the matrix, which reduces the load transfer effect mentioned by others [54,55]. Nevertheless, data show that binder type is a sensitive factor at lower fibre content, while at higher fibre volumes, the strength gain depends mainly on the fibre’s ability to redistribute stress and prevent crack growth [54].

### 3.7. Freeze–Thaw Cycling

**Figure 12:**
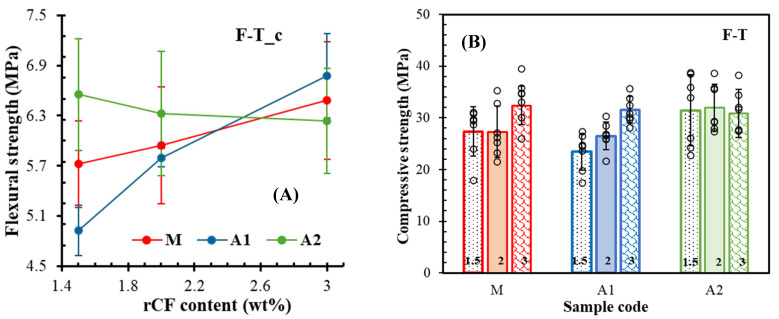
Impact of fibre content on (**A**) flexural and (**B**) compressive strength of mortars after freeze–thawing.

After freeze–thaw cycling, the significance of the binder type becomes more apparent. Figure 12 shows flexural and compressive strength results after freeze–thawing. The results reveal that the type of binder and fibre content are crucial to the frost resistance of composites. Compared with samples hydrated for 28 days (Figure 11A), the flexural strength of all samples reduced by three to 15% after freeze–thawing (Figure 12A). Interestingly, only the A2 set, being the highest group (Figure 11A), showed a weakening effect with an increase in fibre content after freeze–thaw cycling (Figure 12A). Contrastingly, in comparison with 28-day hydrated samples (Figure 8A), the compressive strength (Figure 12B) increased (7–18%) for the sets with intermediate or the highest fibre dosages; however, a few compositions showed weakening (up to 10%) in strength. Nevertheless, the comparison of data obtained from long-term hydration (Figure 8B) reveals that the compressive strength of all sets reduced after freeze–thawing. The reduction in the A1 set is higher than in the A2 group, although the ranges are similar, at 6–15% and 9–13%, respectively. However, the A2 set is less sensitive to fibre content than other compositions. This implies that continuous hydration took place during the freeze-thawing process, resulting in an increase in strength and thus inferring the binder type as a more sensitive factor considering reinforcement with rCF.

### 3.8. Resistance to Water Absorption and Sorptivity

**Figure 13:**
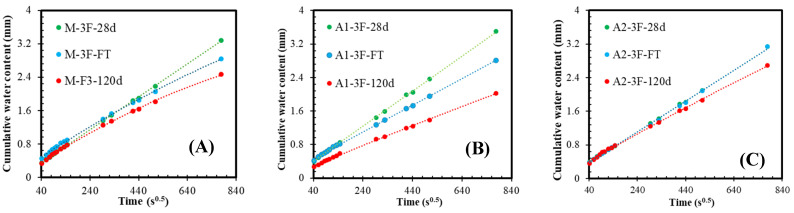
The impact of curing conditions and binder type on the capillary absorption rate of the (**A**) M-F, (**B**) A1-F, and (**C**) A2-F mortars.

**Figure 14:**
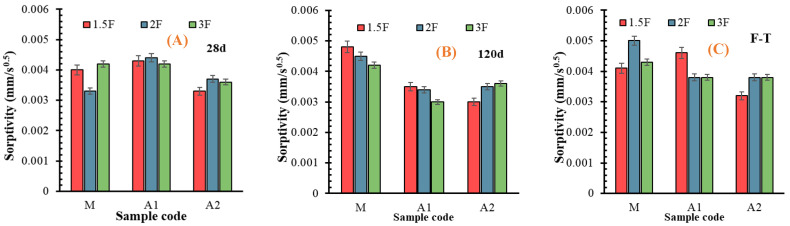
Sorptivity of fibre-reinforced mortars after (**A**) 28 days, (**B**) 120 days, and (**C**) freeze–thawing.

The density of cement-based material depends on the compactness of hydration products, which reduces the structure’s porosity and obstructs water’s ingress into deeper layers [83]. Microcapillary pores (less than 50 nm) are mainly involved in capillary suction, whereas large pores or voids contribute little to capillary transport [51]. However, they may increase the average path of water migration and, thus, slow down the water ingress. On the other hand, recovered carbon fibres (rCFs) serve as micro-reinforcements, as their diameter ranges from 10 to 20 µm, while their length is less than 10–15 mm. However, it was shown in [83,84] that incorporating fibres of different origins increases the ingress rate due to poor interfacial bonding between fibre and cement matrix. Figure 13 shows the water intake rates for samples with the highest dosage of rCF. The linear increment is seen for all groups after 28 days and after freeze–thawing for A1 and A2. As the hydration time increases, water absorption gradually declines from a linear relationship. Although the water ingress rate is very similar among samples, the A2 group is the least dependent on the curing conditions. The sorptivity coefficients, calculated from the linear part of the curves obtained during the first six hours after immersion, are summarised in Figure 14. In general, the sorptivity data are irrelevant to the trend of compressive strength (Figure 8 and Figure 12B), except for the reference group with intermediate fibre content after the freeze–thawing test. The data in Figure 14 reveals that the impact of fibre content is marginal. However, the effect of binder type is important, as the sorptivity of the A2 group remains at the same level after all curing conditions. In contrast, the A1 set, having the lowest strength, exhibits sorptivity comparable to that of the A2 set after long-term hydration and freeze–thawing. Moreover, the freeze-thawing had an insignificant effect on the sorptivity of both sets A1 and A2 compared with long-term hydration. It is worth mentioning that the sorptivity of compositions without MK was up to 40% higher [9]. This implies that the contribution of chemical reactions between ash and MK changes the content of hydration products and increases the contact area between rCF and matrix [82].

### 3.9. Environmental Impact Assessment

**Figure 15:**
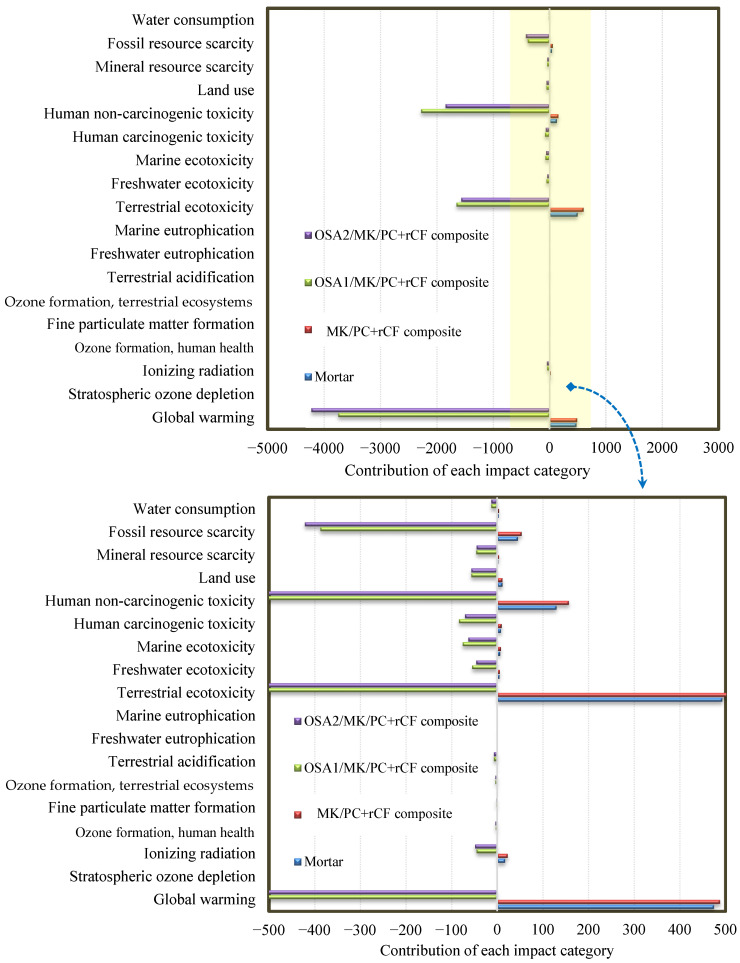
Distribution of impact categories for each production scenario.

The environmental impact categories of mortar, OSA1/MK/PC, and OSA2/MK/PC + rCF composite production are summarised in Table 5. Their contributions are presented in Figure 15. A slight increase in environmental impacts was observed when mixing mortar with OSA and MK additives (MK/PC + rCF composite scenario) by up to 32%. Mineral resource scarcity increased by up to 73%. This is due to the use of plasticisers, which were absent in the case of mortar, as well as the energy consumed in producing MK (mineral source) by kaolin calcination. In the case of the OSA/MK/PC + rCF composite, very promising results were obtained in all categories compared to the mortar and MK/PC + rCF composite scenarios. These included global warming (−3749 and −4225 kg CO_2_ eq), terrestrial ecotoxicity (−1651 and −1567 kg 1,4-DCB), human non-carcinogenic toxicity (−2280 and −1844 kg 1,4-DCB), and fossil resource scarcity (−387 and −422 kg oil eq). These significant reductions resulted from eliminating the sending of rCF to landfills and avoiding its emissions. Additionally, the amount of cement used in composite materials was significantly reduced. Furthermore, the total mass of the composite decreased significantly from 2233 kg (mortar) to 1830 kg (OSA2/MK/PC + rCF). This reduced the energy required to produce its components and its emissions as well. Finally, it was noted that the composition of OSA does not affect the environmental impacts of the composite. Based on these results, OSA, MK, and rCF have promising potential to enhance the environmental performance of mortar. This makes it more environmentally friendly and reduces its environmental burden.

## 4. Conclusions

This study examined the partial replacement of cement with 20% sulfate-rich or carbonate-rich oil shale ash (OSA), combined with 6% metakaolin (MK). Recycled short carbon fibres (rCFs) from the pyrolysis of wind turbine blades (WTBs) were added to advanced cement mortar. Their effects on compressive and flexural strength were monitored during extended hydration and after freeze–thaw cycles. The effects of MK, OSA, and rCF on cementitious phase development, hydration products, specific surface area, mechanical properties, and composite sorptivity were systematically assessed. Life cycle assessment (LCA) was also conducted. XRD analysis showed that interaction with MK increased monocarboaluminate (Mc) in mixtures where cement was replaced with carbonate-rich OSA (A2 group). This led to a refined pore structure and greater volumes of reaction products, primarily calcium silicate hydrates (C-S-H), as confirmed by nitrogen physisorption and thermogravimetric analysis (TGA). The results demonstrated that binder mineralogy, rather than fibre content, most strongly influenced the formation of a dense microstructure, improving water resistance and maintaining mechanical performance after long-term hydration and freeze–thaw cycles. However, a higher rCF content (3 wt.%) proved most effective in producing a strong, frost-resistant composite when cement was substituted by OSA and MK, due to the fibres’ ability to distribute stress and inhibit crack growth. Finally, LCA results indicated that OSA, MK, and rCF have promising potential to improve the environmental performance of mortar across most impact categories.

## Figures and Tables

**Table 1:** Chemical composition of binder components.

Sample	CaO	SiO_2_	Al_2_O_3_	Fe_2_O_3_	TiO_2_	P_2_O_5_	K_2_O	MgO	Na_2_O	SO_3_	MnO	LOI
PC	63.72	13.82	3.85	1.75	0.18	0.08	0.33	1.01	0.23	2.02	0.12	2.66
MK	0.16	47.80	40.1	0.39	1.06	0.51	0.49	-	-	-	-	0.51
OSA1	31.95	23.51	7.54	4.46	0.52	0.18	4.23	4.60	0.42	9.50	0.06	6.42
OSA2	38.77	22.07	7.90	3.93	0.53	0.15	3.84	3.53	0.16	4.52	0.05	9.75

**Table 2:** Compositions and codes of rCF-reinforced cement mortars.

Components	Control (M) Groups			A1 Groups			A2 Groups		
	M-1.5F	M-2F	M-3F	A1-1.5F	A1-2F	A1-3F	A2-1.5F	A2-2F	A2-3F
PC (wt.%)	94	94	94	74	74	74	74	74	74
MK (wt.%)	6	6	6	6	6	6	6	6	6
A1 (wt.%)	-	-	-	20	20	20	-	-	-
A2 (wt.%)	-	-	-	-	-	-	20	20	20
rCFs (wt.%)	1.5	2	3	1.5	2	3	1.5	2	3
Plasticiser (wt.%) of binder content	1.25								
Binder-to-sand ratio	1:3								

**Table 3:** Inventory data required to produce 1 m^3^ (FU) of mortar composites.

Input Materials	Estimated Value per FU		
	Mortar	MK/PC + rCF Mortar	OSA/MK/PC + rCF Mortar (A1-3F and A2-3F)
Portland cement (PC)	496.2	473.11	297.32
Sand	1488.6	1503.36	1200.67
Metakaolin (MK)	----	28.57	22.82
Oil shale ash (OSA)	----	----	80.54
Recycled short carbon fibre (rCFs)	----	9.05	9.05
Plasticiser	----	8.98	10.08
Water	248.1	255.46	209.40
Total mass	2233	2279	1830
Electricity for dry mixing (kwh/FU) [64]	8.93	8.93	8.93
Electricity for wet mixing (kwh/FU) [64]	8.93	8.93	8.93

**Table 4:** Bound water, CH, and CC content of A1 and A2 pastes at different ages from TGA data.

Sample	A1 Paste		A2 Paste	
Hydration time	28 days	120 days	28 days	120 days
Bound water (%)	20.58	21.89	24.66	25.85
Portlandite (%)	12.54	10.67	12.30	15.94
Carbonate (%)	11.05	12.51	14.53	20.43

**Table 5:** Environmental impact categories of production of mortar, OSA/MK/PC, and OSA/MK/PC + rCF composites.

Impact Category	Unit	Mortar	MK/PC + rCF Composite	OSA1/MK/PC + rCF Composite	OSA2/MK/PC + rCF Composite
Global warming	kg CO_2_ eq	474.8	488.6	−3748.7	−4224.7
Stratospheric ozone depletion	kg CFC11 eq	0.0	0.0	0.0	0.0
Ionising radiation	kBq Co-60 eq	17.3	23.0	−45.5	−48.9
Ozone formation, human health	kg NOx eq	0.8	0.9	−3.7	−3.9
Fine particulate matter formation	kg PM2.5 eq	0.3	0.3	−2.5	−2.5
Ozone formation, terrestrial ecosystems	kg NOx eq	0.9	0.9	−4.0	−4.3
Terrestrial acidification	kg SO_2_ eq	0.8	0.8	−7.5	−7.6
Freshwater eutrophication	kg P eq	0.1	0.1	−0.2	−0.2
Marine eutrophication	kg N eq	0.0	0.0	0.0	0.0
Terrestrial ecotoxicity	kg 1,4-DCB	493.5	600.6	−1651.1	−1567.3
Freshwater ecotoxicity	kg 1,4-DCB	4.6	5.8	−54.9	−45.9
Marine ecotoxicity	kg 1,4-DCB	6.2	7.8	−75.8	−63.5
Human carcinogenic toxicity	kg 1,4-DCB	8.3	9.4	−84.0	−70.6
Human non-carcinogenic toxicity	kg 1,4-DCB	129.9	157.4	−2279.6	−1844.4
Land use	m^2^a crop eq	10.9	11.4	−57.3	−57.0
Mineral resource scarcity	kg Cu eq	2.5	4.3	−45.8	−45.3
Fossil resource scarcity	kg oil eq	45.0	52.9	−387.4	−421.7
Water consumption	m^3^	3.1	3.5	−13.5	−13.0

## Data Availability

The original contributions presented in this study are included in the article. Further inquiries can be directed to the corresponding author.
